# A Prospective Study of the Family Quality of Life, Illness Perceptions, and Coping in Mothers of Children Newly Diagnosed with Autism Spectrum Disorder and Communication Difficulties

**DOI:** 10.3390/ejihpe14080146

**Published:** 2024-08-01

**Authors:** Angelos Papadopoulos, Angeliki Tsapara, Alexandros Gryparis, Dionysios Tafiadis, Nikolaos Trimmis, Panagiotis Plotas, Petros Skapinakis, Meropi Tzoufi, Vassiliki Siafaka

**Affiliations:** 1 School of Humanities and Social Sciences, University of Patras, 26504 Patras, Greece; up1110303@ac.upatras.gr; 2 General Children’s Hospital of Patras “Karamandaneio”, 26331 Patras, Greece; 3 Department of Speech and Language Therapy, School of Health Rehabilitation Sciences, University of Patras, 26504 Patras, Greece; up1090680@upatras.gr (A.T.); nicktrimmis@upatras.gr (N.T.); pplotas@upatras.gr (P.P.); 4 Department of Speech and Language Therapy, School of Health Sciences, University of Ioannina, 4th Km National Road Ioannina-Athens, 45500 Ioannina, Greece; alexandros@uoi.gr (A.G.); siafaka@uoi.gr (V.S.); 5 Laboratory of Primary Health Care, School of Health Rehabilitation Sciences, University of Patras, 26504 Patras, Greece; 6 Faculty of Medicine, School of Health Sciences, University of Ioannina, 45110 Ioannina, Greece; p.skapinakis@gmail.com (P.S.); mtzoufi@uoi.gr (M.T.)

**Keywords:** autism spectrum disorder, newly diagnosed children, family quality of life, illness perceptions, coping strategies, prospective study, communication difficulties

## Abstract

(1) Background: This study assesses the impact of mothers’ illness perceptions about autism spectrum disorder and their coping strategies on the family’s quality of life during the initial period following diagnosis and one year afterward. (2) Method: The sample consisted of 53 mothers of children newly diagnosed with autism spectrum disorder and having communication difficulties who completed the following: the Beach Center Family Quality of Life Scale, the Brief Illness Perception Questionnaire, and the Brief-COPE. (3) Results: The findings revealed a moderate family quality of life in the initial assessment and a lack of a statistically significant change one year later. Notably, statistically significant changes were observed in coping strategies, as in the second assessment, and the score in denial and self-blame decreased. Pearson and Eta analyses indicated several correlations between socio-demographic characteristics, illness perceptions, coping strategies, and family quality of life. Multiple regression analysis showed that positive reframing was positively associated with total family quality of life in the initial period following diagnosis and one year afterward, while self-blame was associated with poorer quality of life in the time after diagnosis. Furthermore, the belief about the controllability of the disorder was correlated with better family quality of life one year after the diagnosis. (4) Conclusions: Illness perceptions and coping can be considered as predictors of family quality of life outcomes one year after the diagnosis of autism spectrum disorder. The focus of interventions, apart from controlling the disorder’s symptoms, should aim to strengthen specific strategies and weaken others.

## 1. Introduction

The importance of early detection and focused interventions to improve the functioning of children with autism spectrum disorder (ASD) is a result of many children receiving an ASD diagnosis [1]. Autism pervasively impacts the family and significantly alters the way of life for each family member affected, whereas family caregivers of children with ASD face various everyday life challenges [1].

In the field of autism, there is an increasing interest [2,3,4] in the quality of life (QOL) among family caregivers. However, the literature is limited regarding the perspective of parents of newly diagnosed children. In addition, there is a gap in the literature regarding prospective studies in relation to quality of life among parents of children with ASD, as the majority focus only on a single point-in-time assessment and not on a follow-up period [2,3,4].

### 1.1. ASD’s Impact on Family (Pozo et al., 2011) [5]

Children diagnosed with autism spectrum disorder (ASD) may encounter challenges in developing language abilities and social interaction, as they have difficulties in receptive and expressive language skills in verbal and nonverbal communication [6,7]. Several factors have been identified that influence the quality of life of families with a child with ASD, including the child’s characteristics (e.g., age, severity of ASD symptoms, communication and behavioral difficulties), parents’ characteristics (e.g., gender, perceptions, coping, stigma, psychological discomfort), and many socioeconomic characteristics of the family (e.g., family income, and social support) [5,8,9,10,11,12]. Furthermore, poor mental and physical health, social and a lack of unity within the family lead to a negative impact on the parental well-being [13]. In addition, it is well known that parents face significant difficulties with daily care activities and other challenges (e.g., financial) when raising a child with chronic conditions [14]. Specifically, these families face a lack and pressure of time, increased requirements for parenting, the necessity to offer support for their child’s education, greater expenditures in healthcare, reduced work opportunities, and a higher divorce rate compared to families with typically developing children [15,16]. This results in poor health, psychological distress, exhaustion, and a high risk to the family’s quality of life [17,18] particularly as children grow older and participate in many different social contexts. In particular situations, parents of children with autism may experience a sudden decline in their quality of life, as exemplified by the COVID-19 pandemic [19].

Parents of children with a recent diagnosis of autism are suddenly confronted with a diagnosis, with much information from specialists, and many suggested alternative treatments for their child. At the same time, they are called to accept the diagnosis in a short time, learn to interact with their child, and choose an intervention program for him/her [20,21,22]. In addition, evidence supports that the perceived impact of autism on family quality of life differs between mothers and fathers. Mothers perceived a higher impact of autism on FQoL associated with their child’s internalized disorders (e.g., depressive symptomatology, anxiety), while fathers’ perceptions are correlated with the child’s externalized behaviors (e.g., rule-breaking, aggression) [23]. Parents’ beliefs about autism and the coping strategies they adopt receive growing attention—as cognitive processes mediate people’s adaptation to their health conditions [24].

### 1.2. Parents’ Perceptions about ASD and Coping Strategies

To the best of the authors’ knowledge, there are few studies about the illness perceptions of parents of children with ASD, suggesting that parents’ illness perceptions are associated with their depressive symptoms and their decisions about treatment options [12,18,25]. Parents create their representations to make sense of and manage the challenges related to the diagnosis of ASD. According to the Common-Sense Model [25,26], there is a causal relationship between illness perceptions and illness-related coping strategies and a significant correlation between perceptions and health outcomes and adjustment to illness [27]. According to the literature, parental perceptions have the potential to be altered in the timeline [28], particularly when they pertain to interventions [24,29]. Specifically, when their child is involved in intervention programs, such as speech therapy, occupational therapy, psychotherapy, etc. [24,29]. Finally, it is worth mentioning that most of the studies carried out on parental perceptions toward autism have been cross-sectional in nature [23,30].

Parents adopt coping strategies based on their perceptions of ASD [31]. Coping, according to Lazarus and Folkman, is the mental and behavioral capacity to manage one’s own internal and external demands in the face of stress, and it can be problem- or emotion-focused [32,33,34]. In order to gain a better knowledge of how parents deal with the stress of raising a kid with autism, it is helpful to estimate the coping techniques that parents choose. The coping methods that were utilized by parents of children with autism spectrum disorder who were younger than five years old were significantly different between fathers and mothers [35]. In the literature, traditional gender roles emerge as a feature differentiating the adaption process, as mothers regularly reported utilizing problem-focused and emotion-focused techniques, whereas fathers mainly reported focusing on problem-focused solutions [35]. Parents utilize several coping strategies and resources for successful adaptation, such as direct services to their children, support from spouse and entire family, social support, religion, spirituality, gaining knowledge about the disorder, information exchange, group therapy, nonjudgment interactions, support groups and talking with other families about coping with autism [35,36,37,38]. Nevertheless, the above experiences are not universal, and among others, some cultural factors [33,38] can influence parents’ reactions, resulting in a wide range of outcomes for parents and families [35].

Lutz et al. [36], propose that there is a process of adaptation that mothers go through in response to a series of occurrences that differ from their expectations. The autism diagnosis, often known as “the curveball”, serves as the catalyst for the family’s journey. The model illustrates the impact of a child’s autism diagnosis on the family, including their reactions to the stressor, the diagnosis itself, and the ways they adapt to cope with it. Mothers overcome the challenges of caring for their child or adult with autism by adjusting to their demands, as shown in the arrows and circular form of the model. The progression towards adaptation and the non-linear characteristic of stressor coping are symbolized by the dashed lines surrounding adaptation and between the coping strategies. The mothers’ experiences unveiled an ongoing process of adjustment through the use of coping mechanisms, which started with a quest for solutions in response to feelings of anger and grief. It is a continuous procedure that occurs at various stages in the life of children with ASD as they become older. Mothers exhibit four notable responses to the stressor: grief and anger, illness and relationship strain, guilt and doubt, and disappointment and sacrifice. The narrow, unbroken arrows represented the connections between stressor responses and coping techniques.

A qualitative study of parents of children recently diagnosed with ASD and communication disorders identified nine primary coping mechanisms. These coping mechanisms can be grouped into three categories: (a) adjusting to personal changes, (b) creating treatment strategies for the child with ASD diagnosis, and (c) seeking support [39]. Moreover, based on the results of another study, mothers’ experiences, during the initial and difficult period of diagnosis, include feelings of guilt and blame, acceptance, concentration on the present moment, worry about the future, confusion, competence, isolation, and support [40].

### 1.3. Purpose of the Study

Considering the information presented above, the study aims to contribute to and fill a research void in the literature. Previous research [41,42] has mostly focused on examining the factors that influence FQoL using a cross-sectional design at a single point in time. However, to the best of the authors’ knowledge, there is no evidence about the possible FQoL changes over time among parents of children with ASD who face significant communication difficulties [43]. It is important to understand how parents experience the first crucial period after the diagnostic process and how these experiences and perspectives may change over time as parents may encounter greater challenges. Specifically, the purpose of the current study was to investigate how mothers perceive and cope with their children’s ASD and the core deficits in communication in the initial time after diagnosis (within a maximum of 6 months) (T1), the impact of these factors on the family’s quality of life, and the possible modifications observed after one year (T2).

## 2. Materials and Methods

### 2.1. Participants

The 53 mothers of children with a recent ASD diagnosis were recruited from (a) a children’s hospital, when visited to receive the child’s diagnosis by child psychiatrists, and (b) certain speech therapy and occupational therapy centers. In all cases, the diagnosis was announced in person, and the parents were informed in detail by the child psychiatrist and received support or guidance. In many cases, the child psychiatrist who made the diagnosis announcement was different. Nevertheless, a standard line was maintained.

Only mothers responded to the call and expressed their willingness to participate in the study. It is important to mention that the mothers were the primary caregivers. The researchers set specific inclusion criteria: (a) a recent diagnosis of ASD for the child within 6 months; (b) communication difficulties for the child; (c) no family member with a disability; (d) the participants (parents) should be able to read and complete the questionnaires in the Greek language; and (e) the provision of direct care for the child. The DSM-5 was used to categorize the functioning level of children with ASD. Finally, 58 cases met the inclusion criteria, but two mothers decided to decline participation, and three cases were excluded from the analysis because of missing data. Regarding the follow-up after one year, three mothers decide to withdraw from the study. Something important to mention is that all the children had already been involved in early intervention programs for speech and occupational therapy.

Each participant was provided with information regarding the objectives of the study and the utilization of their data. Written consent was obtained from all participants. The study was conducted in accordance with the Declaration of Helsinki and approved by the Institutional Review Board (or Ethics Committee) of Karamandaneio Children’s Hospital, Patras, Achaia, Greece (approval number: 4173).

### 2.2. Instruments

The study was designed to be prospective. A total of 53 mothers completed the following self-administered questionnaires after they received the appropriate information:

1. The Beach Center Family Quality of Life Scale (FQoL)

This scale was designed to measure the FQoL of children with disabilities [44] and includes 25 questions. Family interaction, parenting, emotional well-being, physical/material well-being, and disability-related support were the five subscales that were included in the Beach Center FQoL. A 5-point Likert scale was used for the scoring and the mean score in overall QoL ranges from 1 to 5. The original study indicated that the psychometric properties had an excellent fit {X2 (5) = 3.41, *p* = 0.63; CFI = 1.00, RMSEA 0.00} and the Cronbach’s α for each subscale ranged from 0.80 to 0.92 [45,46,47]. Moreover, in 2016 the scale was translated and validated into Greek [48].

2. The Brief Illness Perception Questionnaire (Brief IPQ)

The Brief IPQ consists of 9 dimensions, and a Likert scale from 0 to 10 was used to measure the cognitive and emotional representations of the disorder. The nine dimensions of the instrument include consequences, timeline, identity, personal control, treatment control, concerns, coherence, emotional representations, and cause. Regarding the scoring, higher values are an indication of a stronger perception. The Cronbach’s alpha of each subscale ranged from 0.79 to 0.89, and the subscales demonstrated good internal reliability. For the current study, we used the Greek language version, and the changes that were considered necessary for the aims of the study were implemented [27,49].

3. The Brief-COPE inventory

This instrument contains 28 items that are divided into 14 sub-scales for measuring the coping strategies adopted by the participants [50]. The Brief-COPE consists of a 4-point Likert scale; the higher scores represent a more frequent use of each coping strategy. For the purposes of the study, the validated Greek version of this instrument was used [51]. In the original study, the Cronbach’s alpha of each subscale ranged from 0.50 to 0.90 [50]. The reliability (Cronbach’s α) of the Brief-COPE in the present study was 0.736, and the subscales ranged from 0.666 to 0.770.

### 2.3. Statistical Analysis

The researchers conducted a descriptive analysis of the demographic characteristics of the family and their children. The study provided the mean values and standard deviations (mean ± SD) for continuous variables, whereas frequencies were supplied for categorical variables. The mean scores of the dimensions and total scores for all questionnaires and scales were calculated based on the specified criteria and treated as continuous variables. In the Brief-IPQ instrument, the nine dimensions were treated as continuous variables. Regarding the associations between the categorical variables and the Family Quality of Life dimensions, the Eta analysis was used. Furthermore, Pearson’s bivariate correlation coefficient was used to analyze the associations between the dimensions of the Brief-IPQ, Brief-COPE, and FQoL domains during the first assessment and reassessment. The mean value comparison analysis was performed for the same continuous variable in the two-paired sample tests to compare the results of the questionnaires between the initial and final assessments. A multiple regression analysis (theoretical approach and strongly correlated items approach) was performed to determine predictors for the final model. The regression analysis models included the following variables: family monthly income (income 800–2500€, income > 2500€), marital status (married, divorced), Brief-IPQ, and BRIEF-COPE dimensions. Moreover, multiple regression analysis on T2 with independent variables from T1 was conducted. All statistical analyses were performed using SPSS v. 29.

## 3. Results

### 3.1. Characteristics of the Sample

The mean age of the 53 mothers was 39.08 (4.43). The majority of the mothers were married (79.2%), and 47.2% had completed university studies. Regarding the children, 42 were males, and 11 were females. In addition, the mean age of the children was 4.49 (1.57) years, with a range from 2.05 to 8.03. Furthermore, according to DSM-5, 14 children (26.4%) were in level 1, 23 children (43.4%) in level 2, and 16 children (30.2%) in level 3. More demographic characteristics of the study population are presented in Appendix A for the children.

### 3.2. Descriptive Statistics of the Instruments

**Figure 1:**
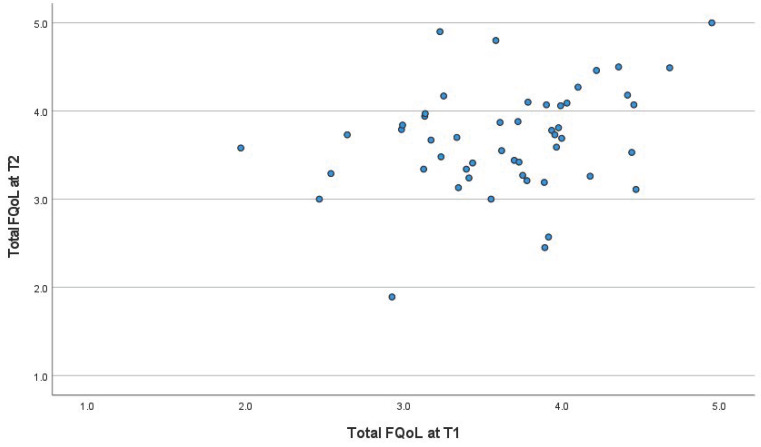
Scatterplot of total FQoL at T1 and T2.

In the initial time after diagnosis (T1), the total mean score on the FQoL was 3.68 ± 0.59. The highest mean score in T1 was observed in the domain “Physical and Material well-being” (3.93 ± 0.79), whereas in T2 it was observed in “Family Interaction” (3.89 ± 0.70). The lowest mean scores were recorded for both T1 and T2 in “Emotional well-being” (3.10 ± 0.82 and 3.19 ± 0.77, respectively), as presented in Table 1. Comparison of the results in T1 and T2 assessment using a paired-samples *t*-test for dependent samples showed no statistically significant difference (Table 1 and Figure 1).

Regarding the Brief IPQ, no statistically significant differences appeared between the two assessments using a paired samples *t*-test for dependent samples. The mean scores in all dimensions of the Brief IPQ are presented in Table 2. It was revealed that in the initial phase after diagnosis (T1), mothers reported strong concerns about the disorder (8.62 ± 1.86) and strong emotional effects of their child’s health condition (8.60 ± 1.74). These perceptions were also strong one year later (T2) (8.60 ± 1.90 and 8.54 ± 1.77, respectively). In addition, in T1, mothers reported an adequate understanding of the disorder’s characteristics (7.83 ± 2.09) and their belief about the effectiveness of therapeutic interventions (7.96 ± 1.74). The reassessment (T2) yielded similar outcomes regarding mothers’ understanding of the disorder (7.96 ± 2.07) and their belief about the controllability of the symptoms through therapeutic interventions (7.94. ± 1.75).

Concerning coping strategies (Brief-COPE) mothers adopted, statistically significant differences appeared between the two assessments. In T1, the coping strategies with the highest mean scores were planning (6.67 ± 1.35), acceptance (6.50 ± 1.38), and active coping (5.92 ± 1.66). In T2 the highest scores were found in acceptance (6.76 ± 1.33), planning (6.66 ± 1.22), and positive framing (6.16 ± 1.65). Statistically significant differences were observed between T1 and T2 in the strategies of denial {t_(49)_ = 2.86, *p* = 0.006} and self-blame {t_(49)_ = 2.52, *p* = 0.015}, as it was observed decrease in their mean score one year after the diagnosis (T2) (Table 3).

### 3.3. Correlations of the Family Quality of Life

The associations between the sample’s demographic characteristics, the children’s clinical characteristics, and the mean scores in the dimensions and total FQoL are presented in detail in Table 4. In T1, negative correlations were found between beliefs about the consequences of the disorder (*p* < 0.001), concerns about the disorder (*p* < 0.010), and emotional representations (*p* < 0.050) with the FQoL total mean score. Conversely, better FQoL in T1 was correlated with higher perceived control over treatment (*p* < 0.001) and a higher coherent understanding of the disorder (*p* < 0.050). Similarly, in T2, better FQoL was associated with stronger perceived control over treatment (*p* < 0.001) (Table 5).

In relation to coping strategies (Brief-COPE) in T1, it was found that the higher denial was correlated with the poorer FQoL (*p* < 0.050). Conversely, better FQoL was correlated with emotional support (*p* < 0.050), positive reframing (*p* < 0.050), and humor (*p* < 0.050). Moreover, in T2, it was revealed that the total FQoL was negatively correlated with denial (*p* < 0.010) and behavioral disengagement (*p* < 0.050), and positively correlated with active coping (*p* < 0.050), informational support (*p* < 0.050), positive reframing (*p* < 0.010), planning (*p* < 0.050), humor (*p* < 0.050), and acceptance (*p* < 0.010) (Table 6).

### 3.4. Multiple Regression Analysis

Multiple regression analysis (Table 7) showed that family monthly income, marital status, denial, self-blame, and positive reframing significantly affected family QoL in the initial assessment. Specifically, married mothers (beta = 0.596, *p* = < 0.001), those who reported high family monthly income (2.500€+) (beta = 0.124, *p* = 0.001), and those who adopted positive reframing as a coping mechanism (beta = 0.166, *p* = < 0.001), had a better FQoL. Conversely, mothers who adopted self-blame as a coping mechanism had a poorer family quality of life (beta = −0.133, *p* = 0.004). Finally, in T2, the multiple regression analysis indicated that total FQoL was affected by positive reframing (beta = 0.107, *p* = 0.028) and perceived treatment control (beta = 0.134, *p* = 0.004). Furthermore, a multiple regression analysis was performed on T2 with the same independent variables as in T1, but no statistically significant results were found.

## 4. Discussion

This study aimed to assess the mothers’ perceptions of ASD, the coping mechanisms they adopted in the initial period after diagnosis (T1) and in the one-year follow-up (T2), and the impact of these factors on the QoL of the family in both time periods.

Regarding mothers’ perceptions of ASD, results revealed no statistically significant difference between the two assessments. In T1 and T2, mothers reported strong concerns about the disorder and beliefs about the emotional implications of their child’s ASD, which is in line with the results of previous articles [24,52,53,54,55,56,57]. Preschoolers whose parents suffer from developmental disorders are more likely to believe that their child’s condition is long-lasting and may lead to depression, according to the available evidence [58], whereas mothers are more likely to develop depressive symptoms during the first years after their child’s diagnosis [27]. The diagnostic process is often described by parents as upsetting, unclear, and confusing, and they typically go through a range of powerful emotions, particularly in the period that follows the diagnosis [28]. Following the diagnosis, the parent’s expectations for their child are associated with the information given about the new situation [29]. Parents who accept their child’s diagnosis and those who can manage their emotions respond better to their parental role and the child’s needs, as “resolving” the diagnosis allows it to be incorporated into an appropriate way of caring [29].

Regarding coping strategies, a comparison between T1 and T2 showed a statistical decrease in denial and self-blame. The relevant literature supports that the feelings of self-blame decreased over time after the diagnosis [59].

This indicates that mothers are more accepting of their child’s situation after one year, which in turn leads to increased adaptation. Denial is the first strategy used by parents before the child’s diagnosis, and it continues in the initial phase [60,61]. During this phase, they are concerned about their child’s behaviors, attributing these concerns to their lack of knowledge about autism [60]. A reduction in self-blame is a stronger predictor of parental adjustment and psychological resilience over time, and the literature supports that feelings of self-blame decrease over 18 months [59].

Generally, it was shown that mothers in the present study used adaptive coping strategies such as planning, acceptance, emotional and informational support, positive reframing, and active coping, findings that are in line with the literature [62]. When a child is diagnosed with a neurodevelopmental disorder, their parents try to understand the disorder through informational support [63,64] and find meaning in the stressful situation through positive reframing, namely thinking about a negative or challenging situation in a more positive way, as well as emotional support from others [39,55,65,66,67].

Regarding the family quality of life (FQoL), the findings from the two assessments indicated that mothers in this study reported a moderate family quality of life even one year after the diagnosis, which aligns with the literature [13,43,47,52,68,69,70,71,72,73,74]. Additionally, the participants seem to be more satisfied with their physical and material well-being and least satisfied with the emotional well-being of their families. With regard to the relationship between the severity of the ASD symptoms and the FQoL, no statistically significant association was observed. Previous studies [75,76,77] have produced inconsistent results regarding the impact of ASD severity on FQoL. While some studies have found that the characteristics of the disorder have a negative effect on FQoL [77], others have suggested that the severity of ASD does not have a significant impact on FQoL [76]. These findings imply that symptom severity may not be the primary factor influencing the well-being and quality of life of parents and other individuals affected by ASD [77]. Thus, it seems that parents may experience increased psychological distress for reasons unrelated to the severity of their children’s ASD symptoms [76].

By comparing the two assessments (T1 and T2), no statistically significant difference in FQoL emerged within one year, although the FQoL was improved in T2. A recent similar longitudinal study conducted in China [43] to examine the characteristics of Family Quality of Life (FQoL) among parents of children newly diagnosed with ASD at two different time points revealed that the overall score of FQoL improved in the follow-up one year after the diagnosis but did not have statistical significance (*p* > 0.05).

Multiple regression analysis revealed that in the initial phase after the diagnosis, monthly family income and marital status can be considered as demographic predictors of the FQoL. Studies among parents of children with ASD support the idea that parents need financial assistance to ensure access to leisure and health, all of which are crucial for a family’s well-being [47,75,78,79]. Families with higher incomes have more alternatives for addressing the increased health and daily needs of family members with ASD [48,67,80,81]. Additionally, having an ASD diagnosis makes parenting even more challenging. As such, being in a two-parent family instead of a single-parent family clearly influenced the family’s quality of life [67,75,82,83]. Furthermore, according to the findings, mothers’ perceptions of treatment effectiveness could be seen as a predictor of FQoL. Participants in the present study seem to have relied on external assistance from therapists, teachers, and services and had higher expectations about improving their children’s symptoms. This could be explained by the fact that all children in the sample participated in intervention programs (e.g., speech therapy, occupational therapy). It has been shown that the availability of support right away following the diagnosis and one year afterward through a well-designed intervention program has a beneficial impact on the parents’ level of life satisfaction [22,84].

Relatively to coping strategies, findings from multiple regression analysis indicated that positive reframing affects FQoL in T1 and T2 [85,86]. Positive reframing involves identifying and replacing maladaptive beliefs with new, more adaptive ones. The mechanism of reframing is not to make a person ignore reality but to give a different meaning to events, as the way a person perceives events determines their thoughts, feelings, and behavior. Recent studies support the idea that focusing on the positive aspects of raising a child with autism significantly contributes to parents’ adjustment to their child’s diagnosis and to responding more fully to the daily challenges of their parenting role. Parents who adopt positive reframing strategies report less stress and better well-being [85,86,87]. Furthermore, it is of note that the literature regarding parental positive perceptions and beneficial aspects of raising a kid with ASD is limited [87,88].

Moreover, findings revealed that low self-blame in T2 was associated with higher scores in FQoL. Studies indicated that caregivers who reported increased self-blame and despair had decreased mental well-being, expressed depressive symptoms, and had lower life satisfaction [59,89,90,91]. These factors also influence parents’ happiness in raising their children [87,89]. It has been observed that self-forgiveness moderates the link between parents’ self-blame for their child’s disability and their well-being, as it is an effective approach for reducing the negative consequences of self-blame [92]. These findings indicate that interventions encouraging self-forgiveness might help parents mitigate harmful consequences linked to self-blame.

It is of paramount importance for health professionals to refer parents in a timely manner to receive specialized psychological interventions. Given that illness perceptions are the outcome of an appraisal process, and the coping strategies represent the ways in which an individual modifies their reactions in order to manage the increased requirements posed by a new situation, it is crucial that the parents have timely access to the appropriate interventions, which can lead to a decrease in the psychological burden and the development of a more positive family environment [85,93]. Determining the illness perceptions and coping strategies that correlate with the quality of life of these families can guide specific intervention programs to strengthen those that appear to function as prognostic factors. Cognitive behavioral therapy interventions, which apply positive reframing as the main therapeutic method, could enhance this coping strategy and help parents become more resilient in the face of challenges posed by ASD, while psychoeducational programs could enhance knowledge about the characteristics of autism and interventions’ effectiveness in improving the functioning of a child diagnosed with ASD. The early involvement of the parents in supportive and training interventions could also improve the interaction with their children and promote the children’s communication skills [85,93].

In conclusion, the findings of the present study demonstrate that family QoL immediately after the diagnosis and one year afterward was at a moderate level, whereas illness perceptions and coping can be considered predictors of FQoL outcome.

### Limitations

This study has some limitations that need to be taken into consideration. Given the inclusion criteria and the prevalence of ASD, it was possible to recruit a relatively small number of mothers; consequently, the findings can only be generalized to a certain extent due to these constraints. The sample consisted of female participants, as they only expressed interest in participating in the study and were the primary caregivers for the children with ASD in the study. It is widely recognized that women are more likely to assume the role of primary caregiver for individuals with physical or mental illnesses [94]. Future studies should focus on including a more representative sample.

Moreover, data from extended long-term follow-ups of the children and their families would be even more important to study the possible changes over time. Finally, another limitation is that the present study did not include participants from rural areas, as the sample consisted of participants from urban environment. There is a lack of research on whether place of residence has an impact on the quality of life of parents who have children with ASD [95]. The potential influence of place of residence may have been disregarded due to its indirect impact, which is influenced by the availability of intervention services. One notable limitation of this study is the problematic Cronbach’s alpha value observed for the Brief-COPE in the original validation, and this might undermine the quality of the results. As far as we know Cronbach’s alpha is a commonly used measure of internal consistency, and a low value can indicate potential issues with the reliability of the scale. However, in this study, the alpha value was 0.736, and the subscales ranged from 0.666 to 0.770, which does not fall below the generally accepted threshold for adequate reliability.

Although our study primarily focused on examining the data independently to address specific research issues and ensure a clear understanding, we recognize the potential advantages of using advanced statistical methods for studying longitudinal data. The latent growth curve model (LGCM) is an approach that offers a comprehensive analysis of individual growth patterns and the factors that impact these changes over time.

However, the study’s most significant contribution is its prospective design and the participation of mothers of children newly diagnosed with ASD due to the existing research gap about the prospective study of the perceptions about the disorder and coping strategies adopted by mothers with a newly diagnosed child with ASD and their impact on the family’s quality of life.

## 5. Conclusions

To develop effective intervention programs for parents of children with ASD, it is necessary to assess the factors that impact FQoL, including the comprehension of the parents’ perceptions about the disorder and the coping mechanisms they adopt, especially in the time following the diagnosis and one year after the initial assessment. To effectively manage the day-to-day challenges they face and take part in decision-making, parents of children with ASD require accurate, comprehensive, and meaningful knowledge regarding the nature of the disorder and the available treatment interventions, as well as well-designed psychological interventions. This would considerably enhance the mental health and quality of life of children and families affected by ASD.

## Figures and Tables

**Table 1:** Comparison of the mean values * of the FQoL scale and its dimensions between T1 and T2.

	FQoL—Τ1		FQoL—Τ2		*p*-Value
	Mean	SD	Mean	SD	NS
Family interaction	3.85	0.75	3.89	0.70	NS
Parenting	3.68	0.66	3.80	0.62	NS
Emotional well-being	3.10	0.82	3.19	0.77	NS
Physical/material well-being	3.93	0.79	3.86	0.73	NS
Disability-related support	3.69	0.71	3.62	0.73	NS
FQoL total score	3.66	0.59	3.68	0.59	NS

* Higher value represented better QoL; FQoL: BEACH Family Quality of Life; SD: standard deviation.

**Table 2:** Scores on the Brief-IPQ-R questionnaire and the comparison of the mean values between T1 and T2.

	Brief IPQ—Τ1		Brief IPQ—Τ2		*p* Value
	Mean	SD	Mean	SD	
Consequences	7.39	2.31	7.54	2.26	NS
Timeline	6.75	2.95	6.72	2.94	NS
Personal Control	6.56	2.24	6.70	2.23	NS
Treatment Control	7.96	1.74	7.94	1.75	NS
Identity	6.90	2.17	6.75	2.21	NS
Illness Concern	8.62	1.86	8.60	1.90	NS
Coherence	7.83	2.09	7.96	2.07	NS
Emotional Representations	8.60	1.74	8.54	1.77	NS
Causes * (frequencies, %)					NS
Emotional cause	5 (9.4%)		7 (13.5%)		
Behavioral cause	10 (18.9%)		12 (23.1%)		
Risk factors	13 (24.5%)		19 (36.5%)		
External factors	12 (22.6%)		14 (26.9)		

Brief-IPQ: Brief Illness Perception Questionnaire; SD: standard deviation; * Perceived cause, as reported by mothers.

**Table 3:** Scores on the Brief-COPE questionnaire and the comparison of the mean values between T1 and T2.

	Τ1		Τ2		Paired Samples Test for Τ1 and Τ2
	Mean	SD	Mean	SD	
Self-distraction	5.01	1.42	5.02	1.53	NS
Active coping	5.92	1.66	6.06	1.36	NS
Denial	4.32	1.59	3.50	1.34	t_(49)_ = 2.86, *p* = 0.006
Substance use	2.03	0.27	2.16	0.58	NS
Emotional support	5.47	1.51	5.46	1.69	NS
Informational support	5.71	1.40	5.56	1.60	NS
Behavioral disengagement	3.07	1.45	2.76	1.15	NS
Venting	5.43	1.47	5.32	1.31	NS
Positive reframing	5.71	1.79	6.16	1.65	NS
Planning	6.67	1.35	6.66	1.22	NS
Humor	2.98	1.16	3.08	1.15	NS
Acceptance	6.50	1.38	6.76	1.33	NS
Religion	4.37	1.96	4.24	1.77	NS
Self-blame	5.54	1.90	4.60	1.67	t_(49)_ = 2.52, *p* = 0.015

Brief-COPE inventory; SD: standard deviation.

**Table 4:** Zero-order correlations between socio-demographic characteristics, clinical characteristics, and FQoL in mothers of children with autism spectrum disorder (ASD).

Family Quality of Life (FQoL)						
	Family Interaction	Parenting	Emotional Well-Being	Physical/Material Well-Being	Disability-Related Support	Total FQoL
Gender of child ^a^	NS	NS	NS	NS	NS	NS
Age of mother ^a^	NS	NS	NS	0.083 *	NS	NS
Education of mother ^a^	NS	NS	NS	0.134 *	NS	NS
Employment status of the mother ^a^	NS	NS	NS	0.190 *	NS	NS
Education of father ^a^	NS	NS	NS	0.227 **	NS	NS
Marital status^a^	0.390 ***	0.167 **	0.129 *	0.238 **	0.268 ***	0.353 ***
Family income ^a^	0.254 **	0.186 *	0.261 **	0.684 ***	NS	0.395 ***
Relevant family medical history ^a^	NS	NS	NS	0.279 **	NS	NS
Attention deficit in children ^a^	NS	NS	NS	NS	NS	NS
2-word phrases time ^a^	NS	NS	NS	NS	NS	NS
Severity ^b^	NS	NS	NS	NS	NS	NS

FQoL: BEACH Family Quality of Life, NS = not significant; ^a^ Eta coefficient test; ^b^ Pearson correlation coefficients; * *p* < 0.05, ** *p* < 0.01, *** *p* < 0.001.

**Table 5:** Correlations between BRIEF-IPQ and the Family Quality of Life Scale (Beach Center FQoL) in the initial assessment (T1) and the reassessment (T2).

Τ1 FQoL						
	Family Interaction	Parenting	Emotional Well-Being	Physical/Material Well-Being	Disability-Related Support	FQoL Total Score
BRIEF IPQ						
Consequences	−0.331 *	NS	−0.434 **	−0.347 *	−0.474 ***	−0.467 ***
Timeline	NS	NS	NS	NS	−0.330 *	NS
Personal control	NS	NS	NS	NS	0.328 *	NS
Treatment control	0.419 **	0.643 ***	0.450 **	NS	0.484 ***	0.556 ***
Identity	NS	NS	NS	NS	NS	NS
Illness concern	−0.344 *	−0.344 *	−0.386 **	−0.293 *	−0.410 **	−0.442 **
Coherence	NS	NS	0.360 **	NS	0.328 *	0.345 *
Emotional representations	−0.374 **	−0.286 *	NS	NS	−0.288 *	−0.328 *
Causes	NS	NS	NS	NS	NS	NS
Τ2 FQoL						
Consequences	NS	NS	NS	NS	−0.346 *	NS
Treatment control	0.503 ***	0.530 ***	0.466 **	NS	0.490 ***	0.516 ***

BEACH Family Quality of Life, BRIEF IPQ: Brief Illness Perception Questionnaire; NS = not significant; Values are Pearson’s correlation coefficients, * *p* < 0.05, ** *p* < 0.01, *** *p* < 0.001.

**Table 6:** Correlations between BRIEF-COPE and FQoL in mothers of children with autism spectrum disorder (ASD).

Τ1 FQoL						
	Family Interaction	Parenting	Emotional Well-Being	Physical/Material Well-Being	Disability-Related Support	FQoL Total Score
BRIEF-COPE						
Active coping	NS	NS	0.305 *	NS	NS	NS
Denial	NS	−0.330 *	−0.298 *	NS	NS	−0.308 *
Substance use	NS	NS	0.281 *	NS	NS	NS
Emotional support	NS	NS	0.273 *	0.366 **	NS	0.282 *
Informational support	NS	NS	0.330 *	NS	NS	NS
Behavioral disengagement	NS	NS	NS	NS	−0.311 *	NS
Venting	NS	NS	0.316 *	NS	0.311 *	NS
Positive reframing	NS	0.324 *	0.305 *	NS	NS	0.301 *
Humor	NS	0.320 *	NS	NS	0.367 **	0.326 *
Religion	NS	NS	NS	NS	0.361 **	NS
Τ2 FQoL						
	Family Interaction	Parenting	Emotional Well-Being	Physical/ Material Well-Being	Disability-Related Support	FQoL Total Score
Active coping	NS	NS	0.344 *	NS	0.394 **	0.323 *
Denial	−0.454 **	−0.431 **	−0.426 **	NS	−0.409 **	−0.439 **
Substance use	NS	NS	NS	−0.363 **	NS	NS
Informational support	NS	NS	0.298 *	NS	0.396 **	0.286 *
Behavioral disengagement	−0.320 *	−0.295 *	NS	NS	−0.300 *	−0.304 *
Positive reframing	0.486 ***	0.381 **	0.350 *	NS	0.476 ***	0.460 **
Planning	0.344 *	NS	0.301 *	NS	0.325 *	0.333 *
Humor	0.303 *	NS	NS	NS	0.329 *	0.289 *
Acceptance	0.391 **	0.423 **	0.321 *	NS	0.368 **	0.402 **

BEACH Family Quality of Life, BRIEF-COPE: The coping orientations to problems experienced. NS = not significant; Values are Pearson’s correlation coefficients, * *p* < 0.05, ** *p* < 0.01, *** *p* < 0.001.

**Table 7:** Statistically significant effects of demographic characteristics, illness perceptions (Brief-IPQ), and coping strategies (Brief-COPE) of the mothers of children with ASD on FQoL for T1 and T2.

		Family Quality of Life (FQoL)—Τ1			
		FQoL Total Score			
		Beta	SE	*p*-Value	95% C.I. for B
R^2^ = 65.0%	Marital status				
	Married	0.596	0.114	<0.001	0.367–0.825
	Monthly income				
	800–2500€	−0.091	0.033	0.010	−0.158–0.023
	2.500€+	0.124	0.029	<0.001	0.066−0.182
	Self-blame	−0.133	0.044	0.004	−0.221–0.044
	Positive reframing	0.166	0.033	<0.001	0.100–0.233
		**Family Quality of Life (FQoL)—Τ2**			
		**FQol Total Score**			
		**Beta**	**SE**	***p*** **-Value**	**95% C.I. for B**
R^2^ = 31.1%	Positive reframing	0.107	0.047	0.028	0.012–0.202
	Treatment control	0.134	0.045	0.004	0.044–0.224

R^2^: R^2^ Adjusted, Beta = unstandardized coefficient B and *p*-values for the family quality-of-life outcomes after hierarchical regression analysis. Results are adjusted for married, divorced, income 800–2500€, income > 2500€, denial, self-blame, and positive reframing. Treatment control. FQoL: Beach Center Family Quality of Life Scale; Brief-IPQ: Brief Illness Perceptions Questionnaire.

## Data Availability

The data that support the findings of this study are available on request from the corresponding author. The data are not publicly available due to restrictions, as they contain information that could compromise the privacy of research participants.

## Appendix A

**Demographic Characteristics of the Families of Children with Autism Spectrum Disorder (ASD) (N = 53)**	
Mothers’ age (years) Mean ± SD (range)	39.08 ± 4.43 (31–49)
Mothers’ age groups	
30–39 (N, %)	30 (56.6)
40–49 (N, %)	23 (43.4)
Marital status (N, %)	
Married	42 (79.2)
Divorced	9 (17)
Unmarried	2 (3.8)
Mother’s educational level (N, %)	
Gymnasium school (preparatory high school)	2 (3.8)
High school	26 (49.1)
University degree	25 (47.2)
Mother’s profession (N, %)	
Economically inactive	21 (39.6)
Farmer	3 (5.7)
Private employee	14 (26.4)
Civil servant	13 (24.5)
Freelancer	2 (3.8)
Relevant family medical history (N, %)	
Developmental disorder	12 (22.6)
Mental disorders	10 (18.9)
Neurological disorder	1 (1.9)
None	30 (56.6)
Monthly family income (euro)	
0–400	6 (11.3)
401–800	15 (28.3)
801–1500	16 (30.2)
1501–2500	10 (18.9)
2500+	6 (11.3)
SD: standard deviation.	

## References

[1] Shu B.C. (2009). Quality of life of family caregivers of children with autism: The mother’s perspective. Autism.

[2] Papadopoulos D. (2021). Mothers’ experiences and challenges raising a child with autism spectrum disorder: A qualitative study. Brain Sci..

[3] Rivard M., Morin D., Coulombe P., Morin M., Mello C. (2022). The Diagnostic Period for Autism: Risk and Protective Factors for Family Quality of Life in Early Childhood. J. Autism Dev. Disord..

[4] Mello C., Rivard M., Morin D., Patel S., Morin M. (2022). Symptom Severity, Internalized and Externalized Behavioral and Emotional Problems: Links with Parenting Stress in Mothers of Children Recently Diagnosed with Autism. J. Autism Dev. Disord..

[5] Pozo P., Sarriá E., Brioso Á. (2011). Psychological adaptation in parents of children with autism spectrum disorders. A Comprehensive Book on Autism Spectrum Disorders.

[6] Kasari C., Brady N., Lord C., Tager-Flusberg H. (2013). Assessing the minimally verbal school-aged child with autism spectrum disorder. Autism Res..

[7] Tager-Flusberg H., Kasari C. (2013). Minimally verbal school-aged children with autism spectrum disorder: The neglected end of the spectrum. Autism Res..

[8] Vasilopoulou E., Nisbet J. (2016). The quality of life of parents of children with autism spectrum disorder: A systematic review. Res. Autism Spectr. Disord..

[9] Dardas L.A., Ahmad M.M. (2014). Quality of life among parents of children with autistic disorder: A sample from the Arab world. Res. Dev. Disabil..

[10] McStay R.L., Trembath D., Dissanayake C. (2014). Stress and Family Quality of Life in Parents of Children with Autism Spectrum Disorder: Parent Gender and the Double ABCX Model. J. Autism Dev. Disord..

[11] Eapen V., Črnčec R., Walter A., Tay K.P. (2014). Conceptualisation and Development of a Quality of Life Measure for Parents of Children with Autism Spectrum Disorder. Autism Res. Treat..

[12] Papadopoulos A., Tafiadis D., Tsapara A., Skapinakis P., Tzoufi M., Siafaka V. (2022). Validation of the Greek version of the Affiliate Stigma Scale among mothers of children with autism spectrum disorder. BJPsych Open..

[13] Reed P., Osborne L.A. (2019). Reaction to diagnosis and subsequent health in mothers of children with autism spectrum disorder. Autism.

[14] Smith S., Tallon M., Clark C., Jones L., Mörelius E. (2022). You Never Exhale Fully Because You’re Not Sure What’s NEXT: Parents’ experiences of stress caring for children with chronic conditions. Front. Pediatr..

[15] Picardi A., Gigantesco A., Tarolla E., Stoppioni V., Cerbo R., Cremonte M., Alessandri G., Lega I., Nardocci F. (2018). Parental burden and its correlates in families of children with autism spectrum disorder: A multicentre study with two comparison groups. Clin. Pract. Epidemiol. Ment. Health.

[16] Karst J.S., Van Hecke A.V. (2012). Parent and family impact of autism spectrum disorders: A review and proposed model for intervention evaluation. Clin. Child. Fam. Psychol. Rev..

[17] Ginieri-Coccossis M., Rotsika V., Skevington S., Papaevangelou S., Malliori M., Tomaras V., Kokkevi A. (2013). Quality of life in newly diagnosed children with specific learning disabilities (SpLD) and differences from typically developing children: A study of child and parent reports. Child. Care Health Dev..

[18] Papadopoulos A., Siafaka V., Tsapara A., Tafiadis D., Kotsis K., Skapinakis P., Tzoufi M. (2023). Measuring parental stress, illness perceptions, coping and quality of life in families of children newly diagnosed with autism spectrum disorder. BJPsych Open.

[19] Pecor K.W., Barbayannis G., Yang M., Johnson J., Materasso S., Borda M., Garcia D., Garla V., Ming X. (2021). Quality of life changes during the covid-19 pandemic for caregivers of children with adhd and/or asd. Int. J. Environ. Res. Public Health.

[20] Suma K., Adamson L.B., Bakeman R., Robins D.L., Abrams D.N. (2016). After Early Autism Diagnosis: Changes in Intervention and Parent–Child Interaction. J. Autism Dev. Disord..

[21] Kent R.G., Carrington S.J., Le Couteur A., Gould J., Wing L., Maljaars J., Noens I., van Berckelaer-Onnes I., Leekam S.R. (2013). Diagnosing autism spectrum disorder: Who will get a DSM-5 diagnosis?. J. Child Psychol. Psychiatry.

[22] Crane L., Chester J.W., Goddard L., Henry L.A., Hill E. (2016). Experiences of Autism Diagnosis: A Survey of over 1000 parents in the United Kingdom. Autism.

[23] Vernhet C., Michelon C., Dellapiazza F., Rattaz C., Geoffray M.M., Roeyers H., Picot M.-C., Baghdadli A., ELENA Study Group (2022). Perceptions of parents of the impact of autism spectrum disorder on their quality of life and correlates: Comparison between mothers and fathers. Qual. Life Res..

[24] Al Anbar N.N., Dardennes R.M., Prado-Netto A., Kaye K., Contejean Y. (2010). Treatment choices in autism spectrum disorder: The role of parental illness perceptions. Res. Dev. Disabil..

[25] Diefenbach M.A., Leventhal H. (1996). The Common-Sense Model of Illness Representation: Theoretical and Practical Considerations. J. Social. Distress Homelessness.

[26] Leventhal H. (1970). Findings and Theory in the Study of Fear Communications.

[27] Karademas E.C., Karamvakalis N., Zarogiannos A. (2009). Life context and the experience of chronic illness: Is the stress of life associated with illness perceptions and coping?. Stress. Health.

[28] Leventhal H., Leventhal E.A., Cameron L., Baum A., Revenson T.A., Singer J.E. (2001). Representations, procedures, and affect in illness self-regulation: A perceptual-cognitive model. Handbook of Health Psychology.

[29] Mire S.S., Gealy W., Kubiszyn T., Burridge A.B., Goin-Kochel R.P. (2017). Parent Perceptions About Autism Spectrum Disorder Influence Treatment Choices. Focus. Autism Other Dev. Disabl..

[30] Dos Anjos B.B., De Morais N.A. (2021). Experiences of Families with Autistic Children: An Integrative Literature Review.

[31] Essex E.L., Seltzer M.M., Krauss M.W. (1999). Differences in coping effectiveness and well-being among aging mothers and fathers of adults with mental retardation. Am. J. Ment. Retard..

[32] Lazarus R.S. (1996). The Role of Coping in the Emotions and How Coping Changes over the Life Course. Handbook of Emotion, Adult Development, and Aging.

[33] Lazarus R.S., Folkman S. (1984). Stress, Appraisal, and Coping.

[34] Lazarus R.S. (1993). Further Annual Reviews from Psychological Stress to the Emotions: A History of Changing Outlooks. www.annualreviews.org.

[35] Pepperell T.A., Paynter J., Gilmore L. (2018). Social support and coping strategies of parents raising a child with autism spectrum disorder. Early Child Dev. Care.

[36] Lutz H.R., Patterson B.J., Klein J. (2012). Coping with autism: A journey toward adaptation. J. Pediatr. Nurs..

[37] O’Nions E., Happé F., Evers K., Boonen H., Noens I. (2018). How do Parents Manage Irritability, Challenging Behaviour, Non-Compliance and Anxiety in Children with Autism Spectrum Disorders? A Meta-Synthesis. J. Autism Dev. Disord..

[38] Tarakeshwar N., Pargament K.I. (2001). Religious Coping in Families of Children with Autism. Focus. Autism Other Dev. Disabl..

[39] Lin C.R., Tsai Y.F., Chang H.L. (2008). Coping mechanisms of parents of children recently diagnosed with autism in Taiwan: A qualitative study. J. Clin. Nurs..

[40] Navot N., Jorgenson A.G., Vander Stoep A., Toth K., Webb S.J. (2016). Family planning and family vision in mothers after diagnosis of a child with autism spectrum disorder. Autism.

[41] Feng Y., Zhou X., Qin X., Cai G., Lin Y., Pang Y., Chen B., Deng T., Zhang L. (2022). Parental self-efficacy and family quality of life in parents of children with autism spectrum disorder in China: The possible mediating role of social support. J. Pediatr. Nurs..

[42] Wang H., Hu X., Han Z.R. (2020). Parental stress, involvement, and family quality of life in mothers and fathers of children with autism spectrum disorder in mainland China: A dyadic analysis. Res. Dev. Disabil..

[43] Dai Y., Chen M., Deng T., Huang B., Ji Y., Feng Y., Liu S., Zhang L. (2023). The importance of parenting self-efficacy and social support for family quality of life in children newly diagnosed with autism spectrum disorder: A one-year follow-up study. Autism Res..

[44] Hoffman L., Marquis J., Poston D., Summers J.A., Turnbull A. (2006). Assessing family outcomes: Psychometric evaluation of the beach center family quality of life scale. J. Marriage Fam..

[45] Hoffman C., Rice D., Sung H.Y. (1996). Persons with chronic conditions: Their prevalence and costs. J. Am. Med. Assoc..

[46] Samuel P.S., Rillotta F., Brown I. (2012). Review: The development of family quality of life concepts and measures. J. Intellect. Disabil. Res..

[47] Mello C., Rivard M., Terroux A., Mercier C. (2019). Quality of life in families of young children with autism spectrum disorder. Am. J. Intellect. Dev. Disabil..

[48] Parpa E., Katsantonis N., Tsilika E., Galanos A., Sassari M., Mystakidou K. (2016). Psychometric Properties of the Family Quality of Life Scale in Greek Families with Intellectual Disabilities. J. Dev. Phys. Disabil..

[49] Broadbent E., Petrie K.J., Main J., Weinman J. (2006). The Brief Illness Perception Questionnaire. J. Psychosom. Res..

[50] Charles S. (1997). Carver. You want to measure coping but your protocol’s too long: Consider the brief COPE. Int. J. Behav. Med..

[51] Kapsou M., Panayiotou G., Kokkinos C.M., Demetriou A.G. (2010). Dimensionality of coping: An empirical contribution to the construct validation of the Brief-COPE with a Greek-speaking sample. J. Health Psychol..

[52] Mire S.S., Tolar T.D., Brewton C.M., Raff N.S., McKee S.L. (2018). Validating the Revised Illness Perception Questionnaire as a Measure of Parent Perceptions of Autism Spectrum Disorder. J. Autism Dev. Disord..

[53] Ntre V., Papanikolaou K., Triantafyllou K., Giannakopoulos G., Kokkosi M., Kolaitis G. (2018). Psychosocial and Financial Needs, Burdens and Support, and Major Concerns among Greek Families with Children with Autism Spectrum Disorder (ASD). Int. J. Caring Sci..

[54] Pozo P., Sarriá E. (2014). Prediction of stress in mothers of children with autism spectrum disorders. Span. J. Psychol..

[55] Feinberg E., Augustyn M., Fitzgerald E., Sandler J., Suarez Z.F.C., Chen N., Cabral H., Beardslee W., Silverstein M. (2014). Improving maternal mental health after a child’s diagnosis of autism spectrum disorder: Results from a randomized clinical trial. JAMA Pediatr..

[56] Oprea C., Stan A. (2012). Mothers of Autistic Children. How do They Feel?. Procedia Soc. Behav. Sci..

[57] Gatzoyia D., Kotsis K., Koullourou I., Goulia P., Carvalho A.F., Soulis S., Hyphantis T. (2014). The association of illness perceptions with depressive symptoms and general psychological distress in parents of an offspring with autism spectrum disorder. Disabil. Health J..

[58] Baker B.L., Blacher J., Olsson M.B. (2005). Preschool children with and without developmental delay: Behaviour problems, parents’ optimism and well-being. J. Intellect. Disabil. Res..

[59] Da Paz N.S., Siegel B., Coccia M.A., Epel E.S. (2018). Acceptance or Despair? Maternal Adjustment to Having a Child Diagnosed with Autism. J. Autism Dev. Disord..

[60] Luong J., Yoder M.K., Canham D. (2009). Southeast Asian parents raising a child with autism: A qualitative investigation of coping styles. J. Sch. Nurs..

[61] Ho K.M., Keiley M.K. (2003). Dealing with Denial: A Systems Approach for Family Professionals Working with Parents of Individuals with Multiple Disabilities. Fam. J..

[62] Al-Kandari S., Alsalem A., Abohaimed S., Al-Orf F., Al-Zoubi M., Al-Sabah R., Shah N. (2017). Brief Report: Social Support and Coping Strategies of Mothers of Children Suffering from ASD in Kuwait. J. Autism Dev. Disord..

[63] Carlsson E., Miniscalco C., Kadesjö B., Laakso K. (2016). Negotiating knowledge: Parents’ experience of the neuropsychiatric diagnostic process for children with autism. Int. J. Lang. Commun. Disord..

[64] Twoy R., Connolly P.M., Novak J.M. (2007). Coping strategies used by parents of children with autism. J. Am. Acad. Nurse Pract..

[65] Poslawsky I.E., Naber F.B.A., Van Daalen E., Van Engeland H. (2014). Parental reaction to early diagnosis of their children’s autism spectrum disorder: An exploratory study. Child. Psychiatry Hum. Dev..

[66] Ekas N.V., Lickenbrock D.M., Whitman T.L. (2010). Optimism, social support, and well-being in mothers of children with autism spectrum disorder. J. Autism Dev. Disord..

[67] McAuliffe T., Cordier R., Vaz S., Thomas Y., Falkmer T. (2017). Quality of Life, Coping Styles, Stress Levels, and Time Use in Mothers of Children with Autism Spectrum Disorders: Comparing Single Versus Coupled Households. J. Autism Dev. Disord..

[68] Malhotra S., Khan W., Bhatia M.S. (2012). Quality of Life of Parents having Children with Developmental Disabilities. Delhi Psychiatry J..

[69] Bonis S.A., Sawin K.J. (2016). Risks and Protective Factors for Stress Self-Management in Parents of Children with Autism Spectrum Disorder: An Integrated Review of the Literature. J. Pediatr. Nurs..

[70] Predescu Elena Ş.R., Fitzgerald M., Yip J. (2017). Family Quality of Life in Autism Spectrum Disorders (ASD). Autism—Paradigms, Recent Research and Clinical Applications.

[71] Reed P., Picton L., Grainger N., Osborne L.A. (2016). Impact of diagnostic practices on the self-reported health of mothers of recently diagnosed children with ASD. Int. J. Environ. Res. Public Health.

[72] Pisula E., Porębowicz-Dörsmann A. (2017). Family functioning, parenting stress and quality of life in mothers and fathers of Polish children with high functioning autism or Asperger syndrome. PLoS ONE.

[73] Kuru N., Piyal B. (2018). Perceived social support and quality of life of parents of children with Autism. Niger. J. Clin. Pract..

[74] Hsiao Y.J., Higgins K., Pierce T., Whitby P.J.S., Tandy R.D. (2017). Parental stress, family quality of life, and family-teacher partnerships: Families of children with autism spectrum disorder. Res. Dev. Disabil..

[75] Calonge-Torres M., Reyes A.L., Avendaño E.L., Conducto C.C., Bautista M.L. (2017). Quality of life of parents of children with autism spectrum disorder aged 3 to 18 years living in an urban area. Arch. Dis. Child..

[76] Alenazi D., Hammad S., Mohamed A. (2020). Effect of autism on parental quality of life in Arar city, Saudi Arabia. J. Fam. Community Med..

[77] Özgür B.G., Aksu H., Eser E. (2018). Factors affecting quality of life of caregivers of children diagnosed with autism spectrum disorder. Indian. J. Psychiatry.

[78] Brown R.I., MacAdam–Crisp J., Wang M., Iarocci G. (2006). Family Quality of Life When There Is a Child with a Developmental Disability. J. Policy Pract. Intellect. Disabil..

[79] Veroni E. (2016). Examing Services Available to Greek Parents of Children with Autism Spectrum Disorders (ASD). Camb. Open-Rev. Educ. Res. e-J..

[80] Piovesan J., Scortegagna S.A., De Marchi A.C.B. (2015). Quality of Life and Depressive Symptomatology in Mothers of Individuals with Autism. Psico-USF.

[81] Lu M., Yang G., Skora E., Wang G., Cai Y., Sun Q., Li W. (2015). Self-esteem, social support, and life satisfaction in Chinese parents of children with autism spectrum disorder. Res. Autism Spectr. Disord..

[82] Harper A., Dyches T.T., Harper J., Roper S.O., South M. (2013). Respite care, marital quality, and stress in parents of children with autism spectrum disorders. J. Autism Dev. Disord..

[83] Nealy C.E., O’Hare L., Powers J.D., Swick D.C. (2012). The impact of autism spectrum disorders on the family: A qualitative study of mothers’ perspectives. J. Fam. Soc. Work..

[84] Saggu R. (2016). Parental Perceptions of the Diagnostic Process for Autism Spectrum Disorder in British. Ph.D. Thesis.

[85] Balubaid R., Sahab L. (2017). The coping strategies used by parents of children with autism in Saudi Arabia. J. Educ. Pract..

[86] Al-Oran H., Khuan L., Ying L.P., Hassouneh O. (2022). Coping Mechanism among Parents of Children with Autism Spectrum Disorder: A Review. Iran. J. Child. Neurol..

[87] Higgins L., Mannion A., Chen J.L., Leader G. (2023). Adaptation of Parents Raising a Child with ASD: The Role of Positive Perceptions, Coping, Self-efficacy, and Social Support. J. Autism Dev. Disord..

[88] Pozo P., Sarriá E. (2015). Still stressed but feeling better: Well-being in autism spectrum disorder families as children become adults. Autism.

[89] Abd Latif M.H., Wan Ismail W.S., Abdul Manaf M.R., Abdul Taib N.I. (2023). Factors Influencing Despair, Self-blame, and Acceptance Among Parents of Children with Autism Spectrum Disorder (ASD): A Malaysian Perspective. J. Autism Dev. Disord..

[90] Eaton K., Ohan J.L., Stritzke W.G.K., Corrigan P.W. (2016). Failing to Meet the Good Parent Ideal: Self-Stigma in Parents of Children with Mental Health Disorders. J. Child. Fam. Stud..

[91] Manan A.I.A., Amit N., Said Z., Ahmad M. (2018). The influences of parenting stress, children behavioral problems and children quality of life on depression symptoms among parents of children with autism: Preliminary findings. Malays. J. Health Sci..

[92] Paleari F., Compare A., Melli S., Zarbo C., Grossi E. Self-blame, self-forgiveness and wellbeing among parents of autistic children. Proceedings of the ECP 2015: 14th European Congress of Psychology “Linking Technology and Psychology: Feeding the Mind, Energy for Life”.

[93] Mira Coelho A., da Conceição V. (2021). Predictors in ASD: The Importance of Parents’ Perception. Front. Psychiatry.

[94] Sharma N., Chakrabarti S., Grover S. (2016). Gender differences in caregiving among family—Caregivers of people with mental illnesses. World J. Psychiatry.

[95] Eapen V., Karlov L., John J.R., Beneytez C., Grimes P.Z., Kang Y.Q., Mardare I., Minca D.G., Voicu L., Abd Malek K. (2023). Quality of life in parents of autistic children: A transcultural perspective. Front. Psychol..

